# Activation of PI3K/AKT and MAPK Pathway through a PDGFRβ-Dependent Feedback Loop Is Involved in Rapamycin Resistance in Hepatocellular Carcinoma

**DOI:** 10.1371/journal.pone.0033379

**Published:** 2012-03-09

**Authors:** Quan-Lin Li, Fang-Ming Gu, Zheng Wang, Jia-Hao Jiang, Li-Qing Yao, Chang-Jun Tan, Xiao-Yong Huang, Ai-Wu Ke, Zhi Dai, Jia Fan, Jian Zhou

**Affiliations:** 1 Department of Liver Surgery, Liver Cancer Institute, Zhongshan Hospital, Fudan University, Key Laboratory of Carcinogenesis and Cancer Invasion of Ministry of Education, Shanghai, People's Republic of China; 2 Endoscopy Center and Endoscopy Research Institute, Zhongshan Hospital, Fudan University, Shanghai, People's Republic of China; 3 Shanghai Key Laboratory of Organ Transplantation, Shanghai, People's Republic of China; The University of Hong Kong, China

## Abstract

**Background:**

Rapamycin is an attractive approach for the treatment and prevention of HCC recurrence after liver transplantation. However, the objective response rates of rapamycin achieved with single-agent therapy were modest, supporting that rapamycin resistance is a frequently observed characteristic of many cancers. Some studies have been devoted to understanding the mechanisms of rapamycin resistance, however, the mechanisms are cell-type-dependent and studies on rapamycin resistance in HCC are extremely limited.

**Methodology/Principal Findings:**

The anti-tumor sensitivity of rapamycin was modest *in vitro* and *in vivo*. In both human and rat HCC cells, rapamycin up-regulated the expression and phosphorylation of PDGFRβ in a time and dose-dependent manner as assessed by RT-PCR and western blot analysis. Using siRNA mediated knockdown of PDGFRβ, we confirmed that subsequent activation of AKT and ERK was PDGFRβ-dependent and compromised the anti-tumor activity of rapamycin. Then, blockade of this PDGFRβ-dependent feedback loop by sorafenib enhanced the anti-tumor sensitivity of rapamycin *in vitro* and in an immunocompetent orthotopic rat model of HCC.

**Conclusions:**

Activation of PI3K/AKT and MAPK pathway through a PDGFRβ-dependent feedback loop compromises the anti-tumor activity of rapamycin in HCC, and blockade of this feedback loop by sorafenib is an attractive approach to improve the anti-tumor effect of rapamycin, particularly in preventing or treating HCC recurrence after liver transplantation.

## Introduction

Hepatocellular carcinoma (HCC) is the second most frequent cause of cancer death in men and the sixth leading cause of cancer death in women [1]. Liver resection is still the mainstay of treatment for HCC and provides the consistent long-term survival. However, the resectability is limited by tumor extent, location, or underlying liver dysfunction, with only a minority of HCC being potentially resectable. All these leave liver transplantation rather than liver resection as the only potentially curative option, which increase the possibilities of HCC resection for patients with nonresectable tumor or severe hepatic failure. Despite the total hepatectomy and liver replace, recurrence and metastasis remained the major obstacles to more prolonged survival after liver transplantation for HCC [2]. Thereby, novel therapeutic strategies to prevent recurrence after liver transplantation are needed.

As a mammalian target of rapamycin (mTOR) inhibitor, rapamycin is clinically used as an immunosuppressive drug to prevent graft rejection after liver transplantation. Meanwhile, rapamycin has also shown anticancer properties *in vitro* and animal models [3], [4], [5]. Because the development and recurrence of cancer are among the most serious side effects of traditional immunosuppression [6], rapamycin may be an effective alternative to conventional calcineurin inhibitor-based therapies in high-risk patients, particularly in HCC patients undergoing liver transplantation. It has already been reported that rapamycin-based immunosuppression is associated with increased survival or recurrence-free survival after liver transplantation for HCC in many studies [7], [8].

Although rapamycin and its analogs show anti-tumor activity across a variety of human cancers, rapamycin resistance is a frequently observed characteristic of many cancers and cancer cell lines [9], [10]. Some studies have been devoted to understanding the mechanisms of resistance of rapamycin [9], [10], however, these mechanisms do not necessarily account for all instances of rapamycin resistance and studies on HCC are extremely limited. Only a study showed that the mechanisms of rapamycin resistance in hepatic cells involved alterations of signaling downstream from mTOR and that the mechanisms were highly heterogeneous [11]. Thus, a better understanding of mTOR signaling and the mechanisms of rapamycin resistance will lead to more optimal targeting of this pathway for enhancing the therapeutic efficacy in preventing and treating tumor recurrence after liver transplantation for HCC.

Thus, in this study, we explored the molecular mechanisms of rapamycin resistance *in vitro* and in an immunocompetent orthotopic rat HCC model, and found that the blockade of associated signaling feedback stemming by sorafenib could overcome rapamycin resistance in HCC.

## Materials and Methods

### Reagents and cell lines

Rapamycin was purchased from Sigma Aldrich for *in vitro* experiments, and from Wyeth for *in vivo* experiments. Sorafenib (BAY 43–9006, Bayer Pharmaceutical Corporation) was dissolved in sterile DMSO for *in vitro* experiments, and in Cremophor EL (Sigma) and 95% ethanol (50∶50) for *in vivo* experiments. DMSO was added to cultures at 0.1% (v/v) final concentration as a vehicle control. Primary antibodies, AKT and phosphorylated AKT (p-AKT; S473); ERK1/2 and phosphorylated ERK1/2 (p-ERK1/2; T202/Y204); PDGFRβ and phosphorylated PDGFRβ (p-PDGFRβ; Y1021) were purchased from Cell Signaling Technology. Glyceraldehyde-3-phosphate dehydrogenase (GAPDH) was purchased from Millipore. Anti-mouse CD31 mAb was purchased from BD Pharmingen. Human or rat PDGFRβ small interfering RNA (siRNA), control siRNA, were obtained from Shanghai GenePharma Co. (Shanghai, P.R.China).

Two human HCC cell lines, HCCLM3 and HepG2 (ATCC), and a rat HCC cell line, Morris hepatoma 3924A (MH3924A) cells (German Cancer Research Center Tumor Collection) [8], were maintained in high-glucose DMEM supplemented with 10% heat-inactivated fetal bovine serum, L-glutamine, 100 units/ml penicillin, and 100 µg/ml streptomycin. Cell lines were cultured at 37°C in a humidified incubator in 5% CO_2_.

### MTT assay

The effects of rapamycin or sorafenib on HCC cell growth were determined with the 3-[4,5-dimethylthiazol-2-yl]-2,5–diphenyltetrazoliumbromide (MTT) assay. Cells were seeded into 96 - well flat-bottom plates (2×10^3^/well) and cultured for 24, 48, or 72 h in medium supplemented with different concentration of rapamycin or sorafenib (6 wells/dose), and each experiment was repeated at least three times. After treatments, cells were incubated with MTT (20 µl/well) at 37°C for 4 h, and then 200 µl DMSO was added. The absorbance of individual wells was determined at 570 nm.

### Determination of apoptotic cells

Cells treated with different concentrations of rapamycin or sorafenib for 36 h were collected. Cells were then treated with 5 µl of Annexin V-FITC solution and PI (1 µg/ml; BD PharMingen, San Jose, CA). Dual parameter flow cytometric analysis was performed to determine the percentage of apoptotic cells (Annexin V alone-positive cells), necrotic cells (PI-positive cells), or viable cells (staining negative for Annexin V and PI).

### Western blot analysis

Western blot analysis was performed as previously described [5]. Briefly, total cell lysates were prepared, and proteins were separated by SDS-PAGE, followed by transfer to polyvinylidene difluoride membrane. The membranes were washed, blocked, and incubated with the specific primary antihuman antibodies against p-AKT (1∶1000), AKT (1∶1000), p-ERK1/2 (1∶1000), ERK1/2 (1∶1000), p-PDGFRβ (1∶1000) and PDGFRβ (1∶1000), or GAPDH (1∶5000), followed by incubation with horseradish peroxidase-conjugated secondary antibodies. Proteins were detected by enhanced chemiluminescence assay (Pierce-Thermo Scientific).

### Quantitative reverse transcription-polymerase chain reaction (qRT-PCR)

Cells were plated and treated with rapamycin or sorafenib. The cells were harvested after 24 h, and total RNA was extracted with Trizol Reagent (Invitrogen) according to the manufacturer's protocol. Total RNA was reverse transcribed with RevertAid™ first-strand cDNA synthesis kit (Fermentas). Human and rat PDGFRs mRNA levels were determined by qPCR using SYBR Premix Ex Taq (TaKaRa, Dalian, China) and normalized to human and rat β-actin respectively, using the primers shown in Table S1. Relative gene expression was calculated with the 2^-ΔCt^ method.

### PDGFRβ silencing by siRNA

PDGFRβ siRNA and negative control mismatch sequences were transfected into LM3, HepG2 and MH cells using Lipofectamine™ 2000 (Invitrogen) according to the manufacturer's instructions. The following sense and anti-sense siRNA strands were used: PDGFRβ (human) GAG GGU GAC AAC GAC UAU ATT (sense), UAU AGU CGU UGU CAC CCU CTT (anti-sense); PDGFRβ (rat) GCC AAU GGC AUG GAA UUC UTT (sense), AGA AUU CCA UGC CAU UGG CTT (anti-sense). After 72 h, cells were lysed, and protein was analyzed by Western blot.

### Animal experiments

Male ACI rats (Harlan Inc., Indianapolis, IN, 200–220 g) were maintained in laminar-flow cabinets under specific pathogen-free conditions and a 12-h dark-light cycle. The animals were cared for and handled according to recommendations of the NIH guidelines for care and use of laboratory animals. The Shanghai Medical Experimental Animal Care Committee approved the experimental protocol. Intrahepatic tumor implantation with Morris Hepatoma fragments was performed under aseptic conditions as previously described [4].

The rats were randomly assigned to four groups (each group, n = 9): untreated vehicle group, rapamycin treatment group, sorafenib treatment group, and rapamycin combined sorafenib treatment group. Treatment was initiated on day 10 after tumor implantatio when all groups had tumors averaging 400 mm^3^, which was demonstrated in our preliminary experiment. Rapamycin (2 mg/kg) or sorafenib (30 mg/kg) was given (orally, once daily) in 500 µl by gavage in single agent-treated group. The mice treated with both rapamycin and sorafenib was administered by using the same schedule for each drug as described for the single treatment. The dosage of rapamycin or sorafenib is based on dosages commonly used in murine models [4], [5]. All control mice received an equal volume of carrier solution by gavage.

Tumor growth was monitored each week by ultrasonography from day 17 after tumor implantation as described previously [4]. Ultrasound scans were performed using a commercially available GE Voluson E8 unit. Tumor volume was calculated in cubic millimeters by multiplying the transverse, anteroposterior, and longitudinal dimensions of the masses. The rats were sacrificed at day 38 after tumor implantation. At necropsy, tumor volume was calculated as V = π/6×length×width×height. Lung and lymph node metastasis, as well as peritoneal seeding, were denoted as the visually positive tumor nodules (Fig. S2). For RT-PCR and Western blot analysis, tumor tissues were homogenized in tumor lysis buffer. For immunohistochemical staining, tumors were fixed in paraformaldehyde for 24 h and embedded in paraffin for sectioning.

### Immunohistochemical PCNA staining for tumor cell proliferation

Paraffin-embedded tissue sections of all tumors were prepared and were labeled first with a proliferating cell nuclear antigen (PCNA)-specific monoclonal mouse antibody (DAKO A/S), followed by staining with a HRP-antimouse immunoglobulin antibody (DAKO A/S). The color reaction was visualized with diaminobenzidine, and tissues were counterstained with Mayers hematoxylin. The Proliferation Index was determined by PCNA immunostaining and calculating the number of PCNA-positive cells per total number of cells (hematoxylin-positive plus PCNA-positive cells) in 5 randomly selected fields at ×200.

### Detection and quantitation of apoptosis

The terminal deoxynucleotidyl transferase-mediated dUTP nick end labeling (TUNEL) method was based on the specific binding of terminal deoxynucleotidyl transferase to the 3′-OH ends of DNA, ensuring the synthesis of a polydeoxynucleotide polymer. For this purpose, the In Situ Cell Death Detection kit-Peroxidase (Roche) was used according to the manufacturer's directions.

The capture of the photographs and measurement of positive staining density were as described previously [5]. Briefly, the tissue sections were viewed at ×200 magnification and images were captured. The Apoptosis Index was determined by counting at least 1,000 cells in 5 randomly selected fields,using Image-Pro Plus v6.0 software (Media Cybernetics, Silver Spring, MD).

### Immunohistochemistry

Tissue sections (4-µm) were stained with hematoxylin and eosin for histologic analysis and with the specific primary antihuman antibodies against p-AKT, p-ERK and CD31 for immunohistochemistry. The tissue sections were viewed at ×200 magnification and images were captured. Five fields per section were analyzed, excluding peripheral connective tissue and necrotic regions. For expression intensity of p-AKT and p-ERK, the integrated absorbance and the area in a photograph were measured using Image-Pro Plus v6.0 software (Media Cybernetics, Inc.). A uniform setting of color segmentation was loaded for counting the integrated absorbance of all the pictures, and the mean p-AKT or p-ERK density was calculated as the product of the integrated absorbance/total area. For the microvessel density (MVD), sections were stained with CD31 antibody and a diaminobenzidine reaction system for immunohistochemical assessment of tumor microvessels in five randomly selected fields at 200× magnification. The average number of microvessels was calculated. For the microvessel count, any brown-stained endothelial cell or endothelial cell cluster that was clearly separated from adjacent microvessels, tumor cells, and connective elements was counted as one microvessel, irrespective of the presence of a vessel lumen.

### Statistical analysis

Statistical analysis was performed with SPSS 16.0 software (SPSS, Chicago, IL). Measurement values were expressed as the mean 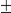 standard error of the mean (SEM). Student's *t* test, χ^2^ test and Fisher's exact test were used as appropriate. Two-tailed p-values<0.05 was judged to be significant.

## Results

### The anti-tumor sensitivity of rapamycin in HCC

In this study, we first examined the sensitivity of rapamycin on the growth and metastasis of HCC.

**Figure 1:**
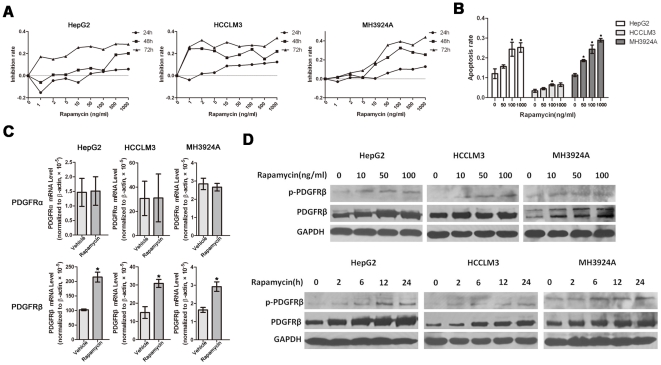
Effects of rapamycin on PDGFR expression and phosphorylation *in vitro*. **A**, Rapamycin inhibited cell proliferation, however, the growth inhibition rate increased slightly even cells were incubated with very high concentration of rapamycin (1000 ng/ml) for 72 h, as assessed by the MTT assay. **B**, Rapamycin induced apoptosis in HCC cells following 24 h treatment, however, the maximal apoptosis rate was only 25.24%, 6.46% and 28.91%, respectively. **C**, Rapamycin significantly up-regulated the mRNA expression level of PDGFRβ but not PDGFRα. After 24-h rapamycin treatment (50 ng/ml), PDGFRα and PDGFRβ mRNA levels were analyzed by qRT-PCR. **D**, The expression and phosphorylation of PDGFRβ were up-regulated by rapamycin in a time and dose-dependent manner as assessed by Western blot analysis. HCC cells were treated with 50 ng/ml rapamycin for different times or with different concentration of rapamycin for 6 h. Three separate experiments were performed in each study. Data are expressed as mean ± SE; *P<0.05, Student's *t* test (versus vehicle).

The HCC cell lines HepG2, HCCLM3, and MH3924A were incubated for 24, 48, and 72 h with rapamycin (0.5–1000 ng/ml). As showed in Fig. 1A, the growth of the above HCC cells were inhibited by rapamycin (minimum effective concentration, 100 ng/ml in HepG2, 10 ng/ml in HCCLM3, and 50 ng/ml in MH3924A for 24 h). However, the growth inhibition rate increased slightly and the maximal growth inhibition rate was only 34.07%, 28.38% and 43.88%, respectively, even cells were incubated with very high concentration of rapamycin (1000 ng/ml) for 72 h. In addition, following 24 h treatment, we showed that rapamycin induced apoptosis in HCC cells (Fig. 1B). However, even cells were incubated with 1000 ng/ml rapamycin, the maximal apoptosis rate was only 25.24%, 6.46% and 28.91%, respectively (basal apoptosis rate, 12.00%, 3.37% and 11.33%, respectively).

We further investigated the anti-tumor effect of rapamycin in an immunocompetent orthotopic rat model of HCC as described previously [12]. As shown in Table 1, although the growth rate of rapamycin-treated tumors was decreased relative to controls, rapamycin treatment alone did not suppress lung metastasis, lymph node metastasis, peritoneal seeding and bloody ascites effectively compared with the vehicle control group (P>0.05 for all).

**Table 1:** The combination therapy enhanced suppression of tumor growth and metastasis in a rodent model of HCC.

Group (n = 9)	Tumor volume(mm3)	Tumor weight (g)	Lung metastasis (%)	Lymph node metastasis (%)	Bloody ascites (%)	Peritoneal seeding (%)
Vehicle	12486.67±2536.88*	13.22±2.17*	9/9(100)#	6/9(66.7)#	5/9(55.6)#	5/9(55.6)#
Rapamycin	5550.90±645.96*	6.47±0.75*	7/9(77.8)#	6/9(66.7)#	4/9(44.4)	2/9(22.2)
Sorafenib	2375.97±312.47*	3.23±0.28*	5/9(55.6)#	3/9(33.3)	0/9(0)	0/9(0)
Combination	788.89±155.37	1.12±0.14	0/9(0)	0/9(0)	0/9(0)	0/9(0)

NOTE: Intrahepatic tumors, lung and lymph node metastasis, bloody ascites, as well as peritoneal seeding in rats were evaluated on day 38 following orthotopic implantation of MH tumor fragments. Treatment with either vehicle or indicated drug (n = 9 per group) started on day 10 after tumor implantation. Tumor sizes were presented by both volume in cubicmillimeters and weight in grams (mean ± SE). Lung and lymph node metastasis, as well as peritoneal seeding, were denoted as the visually positive tumor nodules (the percentages were indicated in parentheses).

* P<0.05, Student's *t* test (versus combination group).

# P<0.05, Fisher's exact test (versus combination group).

Collectively, these data suggested that the anti-tumor sensitivity of rapamycin in HCC was modest.

### Effects of rapamycin on PDGFR expression and phosphorylation *in vitro*

Protein kinases are major regulators of most cellular signaling pathways. Among them, receptor tyrosine kinases (RTKs), such as platelet-derived growth factor receptor (PDGFR), play pivotal roles in promoting cellular growth and proliferation by transducing extracellular stimuli to intracellular signaling circuits. Previous data showed that in Tsc1−/− and Tsc2−/− cells, loss of Tsc1/Tsc2 activated mTOR and down-regulated PDGFR expression; inhibition of mTOR by rapamycin restored PDGFR expression and subsequently enhanced phosphatidylinosital 3-kinase (PI3K)/AKT activation, which reversed the impaired tumor formation by Tsc1−/− and Tsc2−/− cell lines [13], [14]. Given these, we hypothesized that paradoxical up-regulation of PDGFR by rapamycin would also occur in tumor cells, which may impair anti-tumor activity of rapamycin in HCC.

We found that rapamycin (50 ng/ml) significantly up-regulated the mRNA expression level of PDGFRβ after 24 h treatment. In contrast, the mRNA expression level of PDGFRα remained stable during the 24 h treatment (Fig. 1C). Furthermore, we confirmed that the expression and phosphorylation of PDGFRβ were up-regulated by rapamycin in a time and dose-dependent manner as assessed by western blot analysis (Fig. 1D). While this drug at 50 ng/ml, up-regulation and activation of PDGFRβ were evident as early as 2 h after treatment and lasted for 24 h in all HCC cell lines (Fig. 1D). These suggested that PDGFRβ, instead of PDGFRα, was a major negative-regulation target of rapamycin in HCC. Thus, paradoxical up-regulation of PDGFRβ may impair anti-tumor activity of rapamycin in HCC.

### Activation of PI3K/AKT and MAPK pathway through a PDGFRβ-dependent feedback loop is involved in rapamycin resistance *in vitro*

Two of the most important downstream signaling cascades of PDGFRβ are the Ras/Raf/MEK/extracellularsignal-resgulated kinase (ERK) and the PI3K/AKT/mTOR pathways. We next investigated whether up-regulation of PDGFRβ by rapamycin would induce the phosphorylation of AKT and ERK.

**Figure 2:**
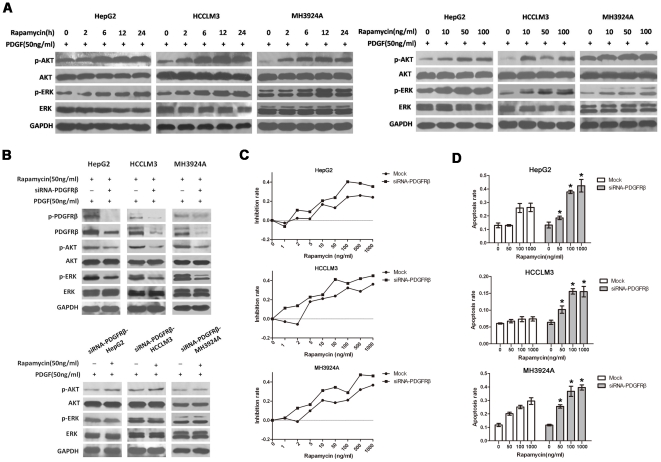
Activation of PI3K/AKT and MAPK pathway through a PDGFRβ-dependent feedback loop compromises the anti-tumor activity of rapamycin *in vitro*. **A**, Rapamycin induced AKT and ERK phosphorylation in a time and dose-dependent manner in response to both rapamycin and PDGF. HCC cells were exposed to both 50 ng/ml rapamycin and 50 ng/ml PDGF for different times, or to different concentration of rapamycin and 50 ng/ml PDGF for 6 h. Proteins were analyzed by Western blot. **B**, PDGFRβ silencing by siRNA inhibited phosphorylation of AKT and ERK in response to both rapamycin and PDGF. HCC cells were exposed to PDGFRβ-targeted siRNA, and then treated with both 50 ng/ml rapamycin and 50 ng/ml PDGF for 6 h. **C**, **D**, PDGFRβ-siRNA significantly enhanced the anti-tumor activity of rapamycin *in vitro*. HCC-siRNA-PDGFRβ cells were incubated with different concentration of rapamycin. The growth inhibition rates were assessed by the MTT assay after 48 h treatment, while the cell apoptosis rates were analysed after 24 h treatment. Three separate experiments were performed in each study. Data are expressed as mean ± SE; *P<0.05, Student's *t* test (versus mock treated with same concentration of ranpamycin).

As shown in Fig. 2A, there were marked augments in AKT and ERK activation in a time and dose-dependent manner in response to both rapamycin and PDGF (50 ng/ml), which corresponded with rapamycin-induced PDGFRβ activation as assessed by western blot analysis. In contrast, total AKT and ERK expression levels were not affected. These results demonstrated that there were significant up-regulations of AKT and ERK phosphorylation in HCC cells in response to both rapamycin and PDGF.

To clarify whether paradoxical phosphorylation of AKT and ERK were PDGFRβ-dependent, we further exposed HCC cells to PDGFRβ-targeted siRNA, and found that siRNA targeted knockdown of PDGFRβ resulted in dephosphorylation of AKT and ERK significantly (Fig. 2B). Similarly, although in response to PDGF, in HCC cells exposed to PDGFRβ-targeted siRNA (HCC-siRNA-PDGFRβ), rapamycin (50 ng/ml for 6 h) did not up-regulate phosphorylation of AKT and ERK prominently (Fig. 2B), suggesting that PDGFRβ-dependent feedback loop may play an important role in rapamycin-induced AKT and ERK phosphorylation in HCC.

Furthermore, we assessed the sensitivity of rapamycin on tumor growth and apoptosis in HCC-siRNA-PDGFRβ cells. Our data showed that the growth inhibition rate and apoptosis rate increased significantly in HCC-siRNA-PDGFRβ cells compared with those in controls (P<0.05 for all, Fig. 2C and 2D). The PDGFRβ-dependent feedback loop was therefore suggested to be responsible for rapamycin resistance in HCC.

### Blockade of PDGFRβ-dependent feedback loop by sorafenib enhances the anti-tumor sensitivity of rapamycin *in vitro* and in an immunocompetent orthotopic rat HCC model

Sorafenib, is a multikinase inhibitor with activity against PDGFRβ and Raf/MEK/ERK signaling pathway. As shown in Fig. S1A and S1B, sorafenib alone inhibited the basal expression of PDGFRβ, as well as the phosphorylation of AKT and ERK significantly. We next evaluated whether sorafenib could deconstruct PDGFRβ-dependent feedback loop and subsequently enhance the anti-tumor sensitivity of rapamycin.

**Figure 3:**
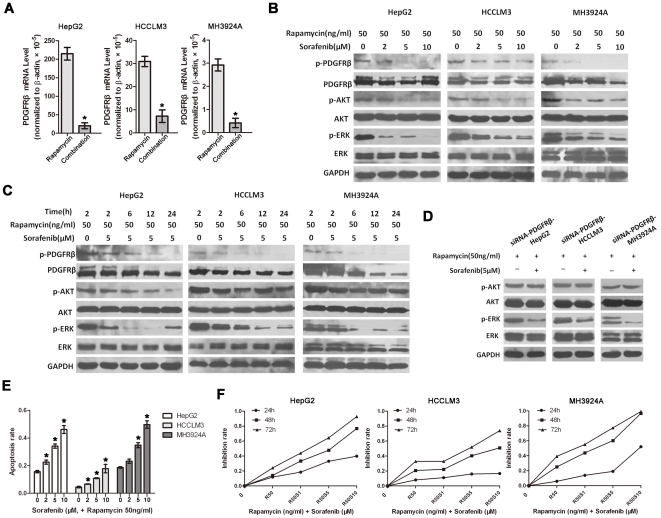
Blockade of PDGFRβ-dependent feedback loop by sorafenib enhances the anti-tumor sensitivity of rapamycin *in vitro*. **A**, As shown by qRT-PCR, sorafenib significantly inhibited the mRNA expression level of PDGFRβ *in vitro*. Cells were treated with rapamycin (50 ng/ml) alone or in combination with sorafenib (5 µM) for 24 h. **B**, **C**, Sorafenib significantly inhibited rapamycin-induced expression and phosphorylation of PDGFRβ, as well as AKT and ERK activation in a time and dose-dependent manner. HCC cells were exposed to both 5 µM sorafenib and 50 ng/ml rapamycin for different times, or to different concentration of sorafenib and 50 ng/ml rapamycin for 6 h. Proteins were analyzed by Western blot. **D**, PDGFRβ-siRNA significantly attenuated the activity of sorafenib against rapamycin-induced AKT phosphorylation. Cells were exposed to PDGFRβ-targeted siRNA, and then treated with 50 ng/ml rapamycin alone or in combination with 5 µM sorafenib for 6 h. **E**, **F**, The combination of rapamycin with sorafenib showed more significant greater effects than rapamycin or sorafenib alone in inhibiting the growth and inducing apoptosis of these HCC cells. cells were incubated with both rapamycin (50 ng/ml) and sorafenib (2, 5, 10 µM), the cell apoptosis rates were analysed after 24 treatment, while the growth inhibition rates were assessed after 48 h treatment. Three separate experiments were performed in each study. Data are expressed as mean ± SE; *P<0.05, Student's *t* test (versus rapamycin alone).

In response to both sorafenib (5 µM for 24 h) and rapamycin, the mRNA level expression of PDGFRβ was significantly decreased in HCC cells (Fig. 3A). As confirmed by western blot analysis, up-regulated expression and phosphorylation of PDGFRβ were also significantly inhibited by sorafenib *in vitro* (Fig. 3B and 3C). In parallel with PDGFRβ inhibition, rapamycin-induced phosphorylation of AKT and ERK were reduced by sorafenib in a time and dose-dependent manner (Fig. 3B and 3C), suggesting that sorafenib was with significant activities against rapamycin-induced phosphorylation of PDGFRβ, AKT and ERK. Furthermore, when HCC cells exposed to PDGFRβ-targeted siRNA, the activity of sorafenib against rapamycin-induced AKT phosphorylation was attenuated evidently (Fig. 3D), suggesting that this inhibition role on AKT phosphorylation may be PDGFRβ-dependent. Collectively, these results suggested that sorafenib could effectively deconstruct the PDGFRβ-dependent feedback loop and subsequently inhibite phosphorylation of both AKT and ERK *in vitro*.

To determine whether that targeted therapy of combining rapamycin with sorafenib showed synergistic anti-tumor effect, we developed experiment to test the consequences of sorafenib individually or together with rapamycin mediated antitumor effects. Our data showed that treatments of these cells with sorafenib alone showed significant growth inhibition and cell apoptosis (Fig. S1C and S1D). Moreover, the combination of rapamycin (50 ng/ml) with sorafenib (1, 5, 10 µM) showed more significant greater effects than rapamycin or sorafenib alone in inhibiting the growth and inducing apoptosis of these HCC cells (Fig. 3D and 3E).

**Figure 4:**
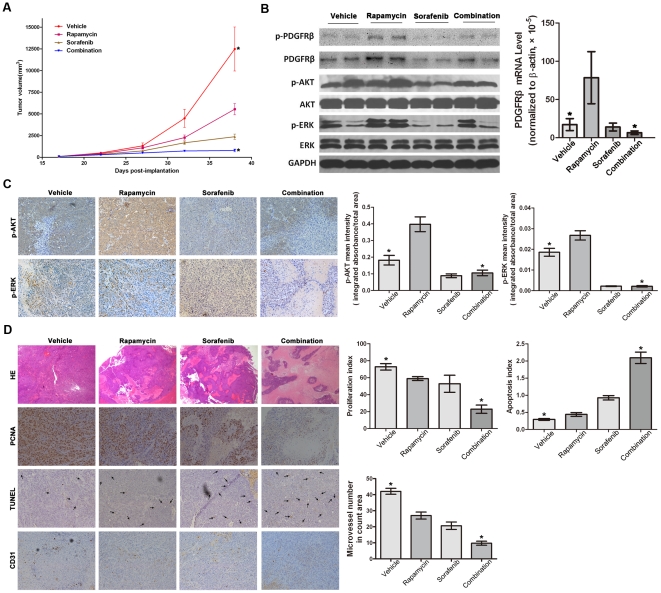
Inhibition of tumor growth and metastasis by rapamycin alone or in combination with sorafenib in an immunocompetent orthotopic rat HCC model. Rats with MH tumors were treated with vehicle, rapamycin (2 mg/kg/d), sorafenib (30 mg/kg/d) or combination (both rapamycin and sorafenib) starting on day 10 after tumor implantation. **A**, The combination therapy showed a significantly stronger suppression of tumor growth rate in comparison with both single agent-treated groups. Tumor growth was monitored each week by ultrasonography from day 17 after tumor implantation. **B**, **C**, RT-PCR, Western blot analysis and immunohistochemistry showed that rapamycin significantly up-regulated the expression and phosphorylation of PDGFRβ, as well as the phosphorylation of AKT and ERK. In combination with sorafenib, rapamycin-induced activation of PDGFRβ, AKT and ERK were significantly inhibited as were *in vitro* results (magnification, ×200). **D**, Significant tumor necrosis in the combination treatment group was visualized by hematoxylin-eosin staining (magnification, ×40). As shown by PCNA and TUNEL, combination treatment also showed more significant greater effects than rapamycin or sorafenib alone in inhibiting tumor cell proliferation and inducing apoptosis of HCC cells (magnification, ×200). The combination therapy also showed significantly fewer tumor vessels than both single agent-treated groups(magnification, ×200). Data are expressed as mean ± SE; *P<0.05, Student's *t* test (versus rapamycin treatment group).

We further investigated the anti-tumor effect of rapamycin alone and in combination with sorafenib *in vivo*. The combination therapy showed not only a much stronger suppression of tumor growth rate, but also significant inhibition of tumor metastasis, in comparison with both single agent-treated groups (Table 1 and Fig. 4A). As shown in Fig. 4B and 4C, rapamycin significantly up-regulated the expression and phosphorylation of PDGFRβ, as well as the phosphorylation of AKT and ERK *in vivo*, suggesting that the PDGFRβ-dependent feedback loop also occurs *in vivo* and plays an important role in rapamycin resistance in this rat model. When in combination with sorafenib, rapamycin-induced activation of PDGFRβ, AKT and ERK were also significantly inhibited as were *in vitro* results (Fig. 4B and 4C). More significant tumor necrosis in the combination treatment group was visualized by hematoxylin-eosin staining (Fig. 4D). As assessed by PCNA and TUNEL staining, combination treatment showed more significant effects than rapamycin or sorafenib alone in inhibiting tumor cell proliferation and inducing apoptosis of HCC cells (P<0.05 for all; Fig. 4D). An additional effect on tumor angiogenesis was also observed in the group that received both drugs. The combination therapy showed significantly fewer tumor vessels than both single agent-treated groups (Fig. 4D).

## Discussion

The immunosuppressive and antitumor effects of rapamycin share a common mechanism of action, thus rapamycin may be an attractive approach for the treatment and prevention of HCC recurrence after liver transplantation. However, the objective response rates of rapamycin achieved with single-agent therapy were modest, supporting that rapamycin resistance is also a frequently observed characteristic of HCC.

Previous studies have shown mechanisms of rapamycin resistance include mutations in FKBP12 and constituents of the mTOR pathway, including S6K1, 4E-BP1, p27kip1, PP2A-related phosphatases [9], [10]. Currently, it is increasingly apparent that mTORC1 pathway is embedded in a network of signaling cross-talks and feedbacks, and rapamycin treatment leads to different signaling responses which may affect its effectiveness in different cell types. In general, it is believed that rapamycin inhibites mTORC1 leading to PI3K/AKT pathway activation through an S6K1-dependent feedback loop in human cancer [15]. Another study showed that rapamycin-induced mitogen-actived protein kinase (MAPK) activation occured in both normal cells and cancer cell lines and that feedback loop depended on an S6K-PI3K-Ras pathway [16]. Recently, studies identified rapamycin-mediated inhibition of PHLPP (PH domain leucine-rich repeats protein phosphatase) expression through a mTOR-dependent compensatory feedback loop may contribute to rapamycin resistance in colon cancer cells [17]. Although substantial body of work has been devoted to understanding the mechanisms of rapamycin resistance, these mechanisms are cell-type-dependent and do not necessarily account for all instances of rapamycin resistance. The mechanisms of rapamycin resistance in HCC are extremely unknown and mandate specific investigation.

The PDGFR is a member of the family of transmembrane receptors with kinase activity and widely recognized as potential targets for anticancer therapeutics [18]. Tumor growth can be promoted through PDGFR by autocrine PDGF stimulation, by overexpression or hyperactivation of PDGFR, or by PDGF stimulation of angiogenesis within the tumor [19]. Previous data showed that inhibition of mTOR by rapamycin up-regulated PDGFR expression in Tsc1−/− and Tsc2−/− cells, and subsequently enhanced PI3K/AKT activation, which reversed the impaired tumor formation by Tsc1−/− and Tsc2−/− cell lines [13], [14]. In this study, our finding that rapamycin significantly up-regulated the expression and phosphorylation of PDGFRβ in HCC cells was partly consistent with these prior studies, whereas the expression of PDGFRα was not affected by rapamycin, suggesting that only PDGFRβ, instead of PDGFRα, was the major negative-regulation target of mTOR and paradoxical up-regulation of PDGFRβ may contribute to rapamycin resistance in HCC.

It remains uncertain precisely how desactivated mTOR up-regulated PDGFR expression. Previous data suggested that up-regulation of PDGFR likely occured due to increased transcription and/or mRNA half-life [14]. The PDGFRβ promoter is controlled by NF-Y and receives input from Myc, Sp1, and p73 [20], [21], [22], suggesting potential roles for these proteins as intermediates in transcriptional promotion consequent to mTOR desactivation. As S6K is significantly dephosphorylated downstream of mTOR and directly contributes to increased IRS activation in response to rapamycin, it is possible that S6K desactivation may directly target some of these regulators of PDGFR expression, leading to increased transcription [23].

PDGFRβ achieves its effects due to activation of various intracellular signaling pathways. Here, we demonstrated that there were major up-regulations of AKT and ERK phosphorylation in HCC cells in response to both rapamycin and PDGF. Using siRNA-mediated PDGFRβ knockdown, we clarified that the PI3K/AKT and the Ras/Raf/MEK/ERK pathways were the specific downstream signaling cascades of PDGFRβ in HCC cells treated with rapamycin. Previously, rapamycin has been reported to lead to PI3K/AKT and MAPK pathway activation through an S6K1-dependent feedback loop in normal cells, as well as in other cancer cells [10], [11]. In this study, our data further extended these findings by indicating that activation of PI3K/AKT and MAPK pathway depended on the paradoxical up-regulation of PDGFRβ in HCC. To the best of our knowledge, this study is the first to demonstrate that activation of PI3K/AKT and MAPK pathway through a PDGFRβ-dependent feedback loop plays an important role in rapamycin resistance in HCC. The identification of this feedback-signaling network will lead to more optimal targeting for the systemic therapy of HCC [24].

As a multikinase inhibitor, sorafenib has shown anti-tumor activity by decreasing ERK and PDGFRβ activation [25], as well as AKT phosphorylation [12] in a group of human malignancies. We found that sorafenib could effectively inhibite phosphorylation of both AKT and ERK in a PDGFRβ-dependent manner, and subsequently enhance the anti-tumor sensitivity of rapamycin *in vitro* and in an immunocompetent orthotopic rat HCC model. Consistent with several recent publications regarding the synergistic anti-tumor activity of rapamycin and sorafenib [5], [26], [27], our findings further demonstrate a novel mechanism that sorafenib enhances the anti-tumor effect of rapamycin through blockade of this PDGFRβ-dependent feedback loop.

We have already shown that rapamycin-based immunosuppression is associated with increased survival or recurrence-free surviva after liver transplantation for HCC [28], [29]. Rencently, partial success of mTOR inhibitors including rapamycin alone or in conjunction with sorafenib also supports the pivotal role of mTOR signalling in the treatment of hepatic and extra-hepatic hepatocarcinoma recurrence after liver transplantation [30], [31]. Our findings provide a better understanding of the mechanisms of rapamycin resistance which may lead to more optimal targeting of this feedback stemming for the prevention and treatment of tumor recurrence after liver transplantation for HCC. However, these warrants further investigation in clinical randomized trials.

**Figure 5:**
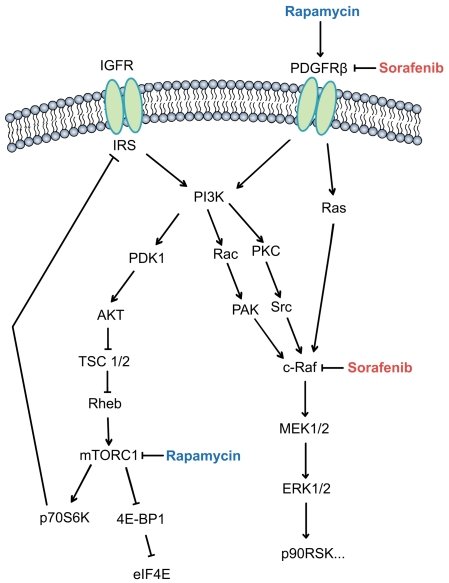
The PDGFRβ-dependent feedback loop compromises the anti-tumor activity of rapamycin in HCC. In HCC, PDGFRβ is the major negative-regulation target of mTOR. Rapamycin paradoxically up-regulates the expression and phosphorylation of PDGFRβ, then subsequently induces activation of PI3K/AKT and MAPK pathway. This PDGFRβ-dependent feedback loop compromises the anti-tumor activity of rapamycin, and blockade of this feedback loop by sorafenib enhances the anti-tumor sensitivity of rapamycin.

In conclusion, our findings confirmed that activation of PI3K/AKT and MAPK pathway through a PDGFRβ-dependent feedback loop compromises the anti-tumor activity of rapamycin in HCC. Moreover, the combination of rapamycin and sorafenib exerts synergistic anti-tumor activity against tumor recurrence and metastasis through blockade of this feedback loop (Fig. 5). Our findings provide a rational basis for combination strategy of rapamycin and sorafenib to maximize the HCC response, particularly in preventing or treating HCC recurrence after liver transplantation.

## Supporting Information

Figure S1 **Effects of sorafenib alone on the growth and apoptosis of HCC cells in vitro.** A, Sorafenib inhibited the basal expression of PDGFRβ. After 24-h sorafenib treatment (5 µM), PDGFRβ mRNA level was analyzed by qRT-PCR. B, Sorafenib inhibited AKT and ERK activation, as assessed by Western blot. HCC cells were exposed to different concentration of sorafenib for 6 h. C, Sorafenib alone inhibited cell proliferation in time and dose-dependent manners, as assessed by MTT assay. D, Sorafenib induced apoptosis in HCC cells following 24 h treatment. Three separate experiments were performed in each study. Data are expressed as mean ± SE; *P<0.05, Student's *t* test (versus vehicle).
(TIF)

Figure S2 **Representative photos of transplanted tumor, regional invasion and lung metastasis.** Arrows point to the transplanted tumor or metastatic focus.
(TIF)

Table S1 **Sequence of primers for RT-PCR.**
(DOC)
