# Supplementary figures and images for: Activation of PI3K/AKT and MAPK Pathway through a PDGFRβ-Dependent Feedback Loop Is Involved in Rapamycin Resistance in Hepatocellular Carcinoma
Source: PLoS One. 2012 Mar 9;7(3):e33379. doi: 10.1371/journal.pone.0033379 (PMC3302853; doi:10.1371/journal.pone.0033379)

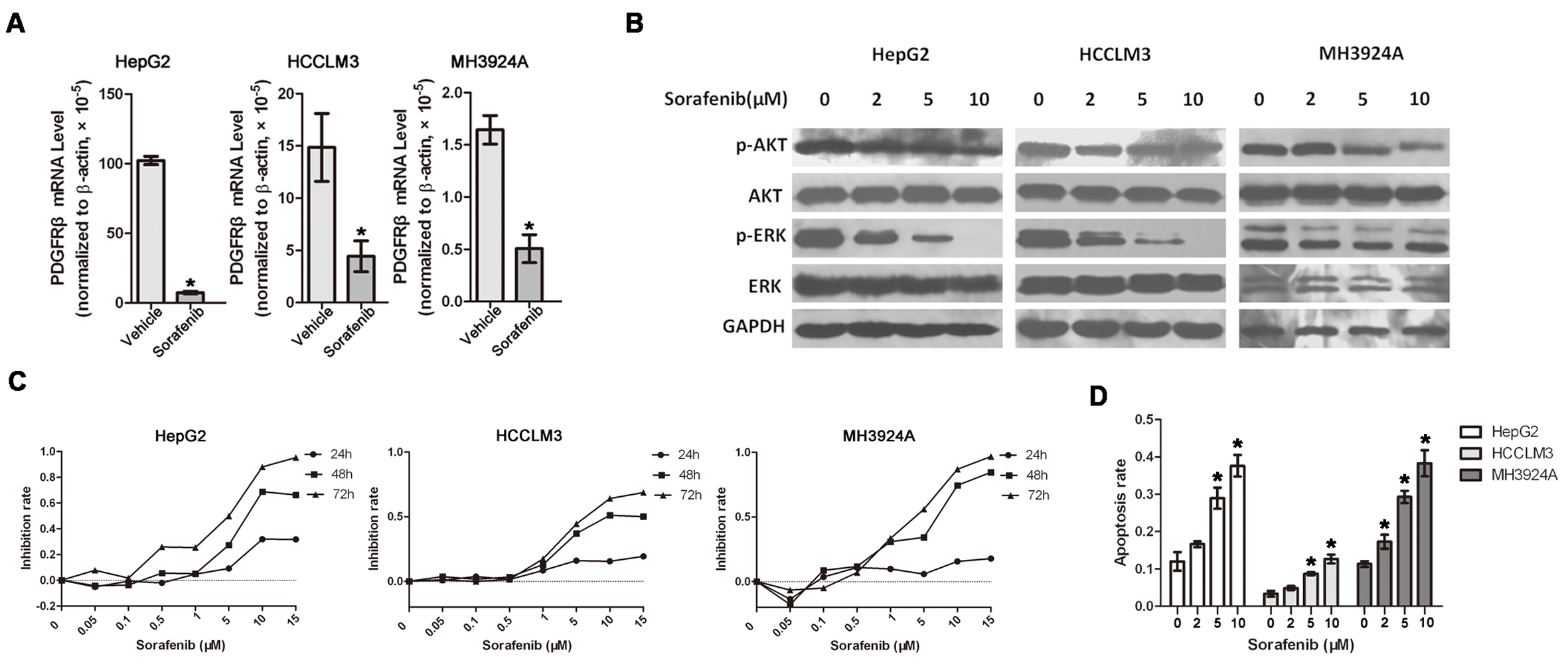

Supplement: Figure S1 — Effects of sorafenib alone on the growth and apoptosis of HCC cells in vitro. A, Sorafenib inhibited the basal expression of PDGFRβ. After 24-h sorafenib treatment (5 µM), PDGFRβ mRNA level was analyzed by qRT-PCR. B, Sorafenib inhibited AKT and ERK activation, as assessed by Western blot. HCC cells were exposed to different concentration of sorafenib for 6 h. C, Sorafenib alone inhibited cell proliferation in time and dose-dependent manners, as assessed by MTT assay. D, Sorafenib induced apoptosis in HCC cells following 24 h treatment. Three separate experiments were performed in each study. Data are expressed as mean ± SE; *P<0.05, Student's t test (versus vehicle). (TIF) [file pone.0033379.s001.tif]

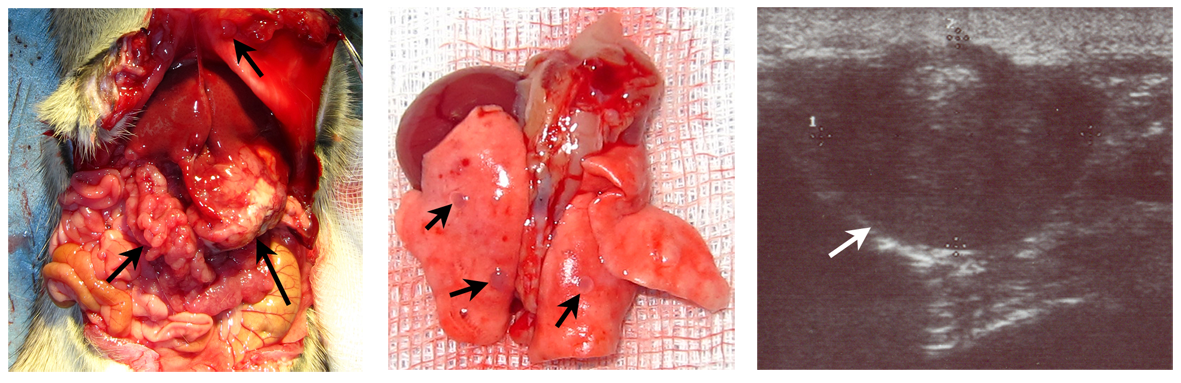

Supplement: Figure S2 — Representative photos of transplanted tumor, regional invasion and lung metastasis. Arrows point to the transplanted tumor or metastatic focus. (TIF) [file pone.0033379.s002.tif]
