# Supplementary material for: Activation of PI3K/AKT and MAPK Pathway through a PDGFRβ-Dependent Feedback Loop Is Involved in Rapamycin Resistance in Hepatocellular Carcinoma
Source: PLoS One. 2012 Mar 9;7(3):e33379. doi: 10.1371/journal.pone.0033379 (PMC3302853; doi:10.1371/journal.pone.0033379)
Supplement: Table S1 — Sequence of primers for RT-PCR. (DOC) [file pone.0033379.s003.doc]

**Table S1. Sequence of primers for RT-PCR.**

| **Gene** | **Forward primer (5’ - 3’)** | **Reverse primer (5’ - 3’)** |
| --- | --- | --- |
| β-actin (human) | CAA CTG GGA CGA CAT GGA GAA AAT | CCA GAG GCG TAC AGG GAT AGC AC |
| PDGFRα (human) | TCT GCC AGC TTT CAT TAC CCT CTA | GTG CCT GCC TTC AAG CTC ATT CTC |
| PDGFRβ (human) | CTG GGC AAA AGG GAC AAA GAG | CAC TGG GCT GGG GAC AAT G |
| β-actin (rat) | TCC ACC CGC GAG TAC AAC CTT CTT | GGC CCG GGG AGC ATC GTC |
| PDGFRα (rat) | GGC GCA GGG GAA AAT CGT GAA G | GGT CGG GTT TGG CCA TCC TGT AT |
| PDGFRβ (rat) | CAG GGC GAG AGC ATC ACC ATC A | CAG CAG CCG CAC ATA GCC ATT TT |
